# Adeno Associated Viral Vector Delivered RNAi for Gene Therapy of SOD1 Amyotrophic Lateral Sclerosis

**DOI:** 10.3389/fnmol.2016.00056

**Published:** 2016-08-02

**Authors:** Lorelei Stoica, Miguel Sena-Esteves

**Affiliations:** ^1^Gene Therapy Center, University of Massachusetts Medical SchoolWorcester, MA, USA; ^2^Department of Neurology, University of Massachusetts Medical SchoolWorcester, MA, USA

**Keywords:** AAV vectors, superoxide dismutase, ALS, RNAi, gene therapy

## Abstract

Amyotrophic Lateral Sclerosis (ALS) is a fatal neurodegenerative disease caused by progressive loss of upper and lower motor neurons. Mutations in superoxide dismutase 1 (*SOD1*) are a leading cause of ALS, responsible for up to 20% of familial cases. Although the exact mechanism by which mutant SOD1 causes disease remains unknown, multiple studies have shown that reduction of the mutant species leads to delayed disease onset and extension of lifespan of animal models. This makes SOD1 an ideal target for gene therapy coupling adeno associated virus vector (AAV) gene delivery with RNAi molecules. In this review we summarize the studies done thus far attempting to decrease *SOD1* gene expression, using AAV vectors as delivery tools, and RNAi as therapeutic molecules. Current hurdles to be overcome, such as the need for widespread gene delivery through the entire central nervous system (CNS), are discussed. Continued efforts to improve current AAV delivery methods and capsids will accelerate the application of these therapeutics to the clinic.

## Introduction

Amyotrophic lateral sclerosis (ALS) is a progressive neuromuscular disorder, resulting in loss of upper and lower motor neurons, leading to paralysis, and death within 3–5 years after diagnosis (Orsini et al., 2015). Mutations in super oxide dismutase 1 (*SOD1*) are responsible for over 20% of familial, or inherited, ALS cases (Renton et al., 2014). Since its discovery as the first ALS causative gene over 20 years ago, *SOD1* has been extensively studied (Rosen et al., 1993). Although the exact mechanism of disease has not been elucidated, *in vitro* and *in vivo* studies have shown that mutations in SOD1 lead to misfolding and aggregation of the mutant protein species (Rakhit and Chakrabartty, 2006; Peters et al., 2015b). Additionally, as SOD1 knockout mice do not develop a paralysis phenotype (Reaume et al., 1996), nor do all SOD1 mutations cause a complete loss of dismutase function, and enzymatically inactive mutant SOD1 still causes motor neuron disease (Borchelt et al., 1994; Wang et al., 2002), it is widely accepted that mutant SOD1 acts through a toxic gain of function mechanism.

A common therapeutic approach for gain of function diseases is reduction of the toxic gene product. Loss of *Sod1* in mice does not lead to paralysis or motor neuron loss. Additionally, there is no survival benefit in expressing mutant human *SOD1* in the presence of mouse *Sod1* compared to *Sod1* null background (Bruijn et al., 1998). This would imply that SOD1 reduction is likely to be a safe therapeutic approach. However, there does appears to be increased motor neuron vulnerability in response to stress and injury in both *Sod1*^−∕−^ and *Sod1*^+∕^^−^ mice (Reaume et al., 1996; Saccon et al., 2013). While this suggests a potential effect due to SOD1 loss of function, complete knockout mouse models are not accurate representation of gene reduction strategies, such as RNAi. In the *Sod1*^−∕−^ mouse, the enzyme is absent from embryonic development, while an RNAi approach would reduce, but not completely eliminate (see below), the levels of an already existing protein. Nevertheless, since the *Sod1*^+∕^^−^ mouse does have a mild phenotype, ideal therapies would be allelic specific, reducing only the mutant gene product. Meanwhile, future clinical trials could consider supplementation with SOD1 enzyme replacement therapy.

Gene therapy for familial ALS has made substantial headway in recent years, especially as the number of preclinical studies and clinical trials using RNAi therapies and AAV vectors have increased rapidly (Borel et al., 2014). Multiple studies have approached SOD1-ALS treatment through the use of AAV delivered RNAi in both mouse and rat models of ALS, as well as target engagement and safety studies in normal monkeys. Although the studies have had various degrees of success, they have consistently shown AAV-RNAi to be a viable therapeutic approach.

## Reduction of *SOD1* is necessary in both neurons and astrocytes

Some of the initial work aimed at assessing the therapeutic potential of reducing mutant SOD1 expression used transgenic mouse models. The most widely used mouse model is the SOD1^G93A^ mouse, which contains multiple copies of the mutated human *SOD1* gene. This mouse reproduces the patient phenotype, including the progressive loss of motor neurons and development of hind limb paralysis. The first study (Saito et al., 2005) crossed mice constitutively expressing an anti-*SOD1* siRNA transgene with SOD1^G93A^ mice, and the double transgenic mice showed no signs of disease. In the study, *SOD1* expression was reduced in all cells, presumably from the first stages of embryonic development. Additionally, the shRNA used was not specific for human *SOD1* but also reduced mouse *Sod1* expression. The absence of a phenotype associated with loss of SOD1 activity in these mice supports the notion that non-allele specific silencing of SOD1 is likely to be safe in humans. A parallel study (Boillee et al., 2006) used conditional knock-out mice where the mutant *SOD1* transgene was flanked by loxP sites. These mice were crossed with other transgenic mice expressing Cre under neuronal or microglial/macrophage promoters, leading to cell type-specific excision of *SOD1*. Both mouse lines displayed an increase in survival, but elimination of *SOD1* in neurons delayed onset, while in glia it slowed disease progression. These two studies in transgenic animals highlighted early on that although motor neurons are selectively vulnerable in ALS, targeting non-neuronal cells is likely crucial to fully treat the disease.

The first gene therapy studies for SOD1-ALS focused on viral vector delivered RNAi molecules to spinal cord motor neurons. The largest increase in lifespan in a SOD mouse model was documented for an approach based on intramuscular injections of a lentivirus encoding a shRNA against *SOD1*. The vector was delivered at postnatal day 7 by direct injection into multiple muscle groups, resulting in a survival increase of 77%, due to a delay in disease onset; suggesting it was successful in silencing SOD1 expression in motor neurons. The muscles injected included the hindlimb, to target lower motor neurons; as well as the facial, tongue, and intercostal muscles, since ALS has not only mobility, but feeding and respiratory deficits as well. While these results were promising, the large number of injections required made clinical translation challenging. It is interesting to note that the researchers chose injections into muscle to take advantage of the retrograde transport capabilities of the (equine infectious anemia virus) EIAV lentivirus vector pseudotyped with the Rabies G envelope glycoprotein, and specifically target the innervating neurons. Thus, it is not surprising that while disease onset was delayed, the length of disease duration was not increased further supporting the notion that motor neurons are mediators of disease onset, but progression is also dependent on expression of mutant SOD1 in glia (Ralph et al., 2005). Another study used a lentivirus to deliver an shRNA through bilateral injection into the lumbar spinal cord, in 40-day-old SOD1^G93A^ mice. This injection approach was successful in targeting motor neurons in the lumbar spinal cord. However, there was no increase in lifespan, despite a 60% improvement in motor neuron survival (Raoul et al., 2005). These outcomes further emphasize the importance of targeting multiple cell types throughout the spinal cord.

Widespread transduction is likely necessary to completely prevent cell autonomous and non-autonomous toxic mechanisms from triggering a cascade of events that ultimately lead to complete loss of motor function. Reduction of mutant *SOD1* in motor neurons and glia, and possibly also muscle, has been shown to be critical to prevent disease manifestation. The presence of *SOD1* in non-neuronal cells is important for disease progression and multiple studies have shown that motor neurons are selectively vulnerable to astrocyte-mediated toxicity (Julien, 2007). Studies in different SOD1 mouse models have shown that reduction of *SOD1* specifically in astrocytes leads to an increase in survival (Boillee et al., 2006; Yamanaka et al., 2008). Additionally, astrocytes from SOD1 mice are toxic to motor neurons, both *in vivo* and *in vitro* (Nagai et al., 2007; Di Giorgio et al., 2008; Haidet-Phillips et al., 2011). An increase in astrocytosis is also associated with *SOD1* astrocyte driven disease progression. A recent study aimed to definitively answer the question as to whether neurons or astrocytes alone can be therapeutic targets using a gene transfer approach. AAV vectors encoding *SOD1*-specific artificial microRNAs (amiRNAs) under cell specific promoters were infused into the lateral ventricles of neonate SOD1 mice (Dirren et al., 2015). An AAV6 vector carrying the cytomegalovirus (CMV) promoter (shown to have strong propensity to transduce motor neurons with this type of serotype and delivery method Dirren et al., 2014) extended survival by 26% while an AAV9 vector carrying a GFAP glial promoter increased survival by 14%. Unfortunately, the combined injection of both AAV vectors showed no additional benefit, and fell short of the large increase in lifespan documented with the intravenous delivery of AAVs encoding shRNA or amiRNA under ubiquitous promoters. However, this could be due to the need to half the dose of both vectors for combined infusion, in order to maintain the same injection volume. Studies are also underway testing a single bicistronic AAV vector where the therapeutic artificial miRNA is expressed from both the neuronal synapsin 1 and glial GFAP promoters. The bicistronic AAV vector appears to have superior therapeutic effect based on motor performance compared to AAV vectors targeting a single cell type (Bobela-Aebischer et al., 2016).

## Therapeutic success of systemic AAV-RNAi delivery is age dependent

The discovery that AAV9 is capable of crossing the blood brain barrier after systemic administration opened a new avenue for development of a gene therapy approach capable of tackling ALS in a global manner (Foust et al., 2009). The first AAV9 based study used an H1 promoter driven shRNA (Foust et al., 2013) and reported a 39% increase in median survival when delivered to SOD1 mice at postnatal day 1 (P1), and 30% when delivered at postnatal day 21 (P21). The spinal cord transduction profile of AAV9 injected systemically changes from motor neurons in neonates to mostly glia in older animals (Foust et al., 2009). Consistently P1 injections led to transduction of more motor neurons (64%) than glia (34%), while it was the reverse for P21 injection with transduction of fewer motor neurons (8%) than glia (54%). The degree of SOD1 protein reduction in the spinal cord was also age dependent with 60% for P1, but only by 45% after P21 injection. In this study, mice were also injected at 85 days of age, when disease symptoms are starting to manifest, leading to a 23% increase in survival. These results raise the question of how late in disease progression is an AAV-RNAi intervention likely to have a therapeutic effect. This has been further confirmed by a recent study (Borel et al., 2016), which also used an intravenous infusion, of AAVrh10, to deliver an artificial microRNA specific to *SOD1*. Mice were treated at 56–68 days, when early signs of disease are already apparent at the molecular and biochemical levels. Testing therapeutic efficacy in early symptomatic adult mice is more relevant compared to neonatal or P21 interventions since ALS patients are diagnosed as adults and often at early stages of disease progression. Treatment with AAVrh10-amiRNA reduced *SOD1* mRNA in transduced cells and increased lifespan by 21%, an outcome similar to the previous treatment initiated at 85 days of age. Since AAV9 (and AAVrh10) target mostly glia after an postnatal day 1 intravenous infusion (Zhang et al., 2011; Yang et al., 2014), it is clear that SOD1 reduction in glial alone is not sufficient, as shown in previous studies with conditional knock-out SOD1 mice (Boillee et al., 2006), and it is likely that more motor neurons need to be transduced to achieve greater therapeutic efficacy. Thus, while an intravenous infusion is the easiest delivery method, current AAV vectors are not efficient enough at crossing the blood brain barrier in adult mice to transduce the majority of cells in the CNS.

## Upper motor neurons, as well as muscle, play a role in disease progression

It has been hypothesized that ALS is caused by a “dying back” mechanism, where degeneration starts in muscle, travels up the axons to the spinal cord motor neurons and then to the cortical layer V motor neurons in the brain. AAV6 has been shown to efficiently transduce skeletal muscle as well as undergo retrograde transport to motor neurons after intramuscular injection (Kaspar et al., 2003; Gregorevic et al., 2004). Thus, several studies used AAV6 to deliver an *SOD1*-specific shRNA in SOD1 mice, to reduce the mutant protein in muscle as well as in the central nervous system (CNS). In the first study, adult SOD1 mice received an intravenous infusion of AAV6-shRNA vector resulting in 50% reduction in SOD1 protein in skeletal muscle. However, < 5% of motor neurons were transduced, and there was no apparent reduction in SOD1 levels in the spinal cord (Towne et al., 2008). In a subsequent study (Towne et al., 2011), AAV6-shRNA was injected directly into multiple muscle groups in neonate SOD1 mice, similar to the EIAV lentivirus study, but there was no change in survival. This was surprising as there was efficient retrograde transport of AAV6 to motor neurons as evidenced by the >50% reduction of *SOD1* mRNA level in transduced cells. This discrepancy is potentially due to the lower number of motor neurons transduced in the AAV6 study compared to the EIAV lentivirus study (40% vs. >50% in the lumbar spinal cord, respectively), or differences in shRNA efficacy. In a separate experiment, an shRNA against *SOD1* was delivered by intramuscular injection of a lentivirus vector pseudotyped with the vesicular stomatitis virus glycoprotein (VSV-G) not capable of retrograde transport, or an AAV2 vector capable of retrograde transport. Both vectors reduced SOD1 protein levels in skeletal muscle, but the improvement in motor function was seen solely in the AAV2 treated SOD1 mouse (Miller et al., 2006). However, partial reduction of mutant *SOD1* in muscle using the Cre-Lox system had no effect on survival (Miller et al., 2006), and expression of mutant *SOD1* in muscle alone led to motor impairment caused by alterations in neuromuscular junction (Jaarsma et al., 2008; Wong and Martin, 2010).

Recently, more attention has been given to the potential role of cortical layer V (upper) motor neuron degeneration in the disease phenotype. Ozdinler et al. (2011) showed that layer V cortical motor neurons also undergo degeneration in SOD1^G93A^ mice, an observation that reproduces the neuropathological findings in the brain of ALS patients (Saberi et al., 2015). Thomsen et al. (2014) demonstrated the importance of this neuronal population in disease progression and as a key therapeutic target: multiple injections of an AAV-H1-shRNA vector covering the motor cortex of SOD1 rats led to a 20-day extension in survival with no evidence of spinal cord transduction. Moreover, this study also raised questions regarding the hypothesis that ALS is caused by a “dying back” mechanism—that disease and degeneration starts in muscle, travels up to the motor neurons of the spinal cord and eventually to those in the brain. Since suppression of the misfolded SOD1 protein in motor cortex resulted in a therapeutic benefit, it is possible that disease starts concomitantly at all levels of the CNS, albeit perhaps with varying kinetics for different susceptible neuronal populations.

## Widespread delivery throughout the CNS remains challenging in mouse models

AAV9 is highly effective for transduction of spinal cord motor neurons, in an age dependent manner, but it remains relatively ineffective for widespread neuronal gene transfer in the brain by systemic delivery. An alternative approach to achieve broader neuronal transduction in the CNS is to infuse AAV vectors into the lateral ventricles of neonatal mice. Stoica et al., performed a proof-of-concept experiment using an AAV9 vector encoding a CBA promoter-driven artificial microRNA specific to human *SOD1*, delivered into the brain lateral ventricles of SOD1^G93A^ mice at post natal day 1 (Stoica et al., 2016). This type of infusion leads to gene expression in cortical and spinal cord motor neurons (Broekman et al., 2006; Chakrabarty et al., 2013; McLean et al., 2014), as well as non-neuronal cells and peripheral muscle. As a result this study reported the longest extension in lifespan to date using AAV mediated RNAi. Interestingly, treated SOD1^G93A^ mice did not die from the typical onset of paralysis, but instead from rapid body weight loss accompanied by a progressively hunched kyphotic posture. A previous study testing the therapeutic efficacy of an AAV6-amiRNA vector delivered by the same route to neonate SOD1^G93A^ mice reported development of similar symptoms unrelated to paralysis (Dirren et al., 2015). The authors of the AAV9 CBA-amiRNA study postulated the cause of death to be a previously undetected respiratory phenotype, similar to the respiratory impairment developed by ALS patients. Indeed, the reported respiratory phenotype in the ALS mice was not fully corrected by AAV treatment, making this a plausible hypothesis to explain the demise of treated mice. It is important to note that although treated mice survived longer with no visible motor impairment, there was evidence of neuronal loss at euthanasia. It is possible that the neurons involved in respiration—in the medulla, cervical, and thoracic spinal cord—were lost in these mice, explaining the observed decrease in respiratory response. Additionally, since this was a neonatal treatment, the therapeutic artificial microRNA would have been lost from post-natally dividing cells in the spinal cord, such as astrocytes. Thus, although this delivery approach was able to achieve widespread transduction, it is still not an accurate representation of the transduction achievable in the clinic as the distribution profile of AAV9 via cerebral spinal fluid (CSF) infusion routes is considerably more restricted in adult animals.

The next step for this therapeutic approach is the translation into adult animals, and larger mammals. The most accessible way to deliver AAVs into the CSF is through intrathecal (IT) infusion into the lumbar spinal cord. This injection route leads to effective transduction of spinal cord motor neurons in adult non-human primates, but the percentage of transduced neurons in the brain remains limited. Studies using intrathecal infusions of AAV vectors in mice reported variable reproducibility, and limited vector spread to distal spinal cord regions and to the brain (Snyder et al., 2011). Wang et al. (2014) performed IT injections of an AAVrh10 vector encoding an artificial miRNA against SOD1 into the lumbar spinal cord of adult SOD1^G93A^ mice. This resulted in an 11% average increase in survival and a direct correlation between survival and the level of transduction of the spinal cord. Patel et al. performed IT injections of an AAV1 encoding a single chain antibody against misfolded SOD1 and reported a 28% increase in survival of treated SOD1^G93A^ mice. However, survival was highly variable among treated animals, which directly correlated with antibody titers in the spinal cord (Patel et al., 2014). This highlights the still unsolved problem of widespread AAV delivery to motor neurons in adult mice. Multiple studies have shown effective transduction of both motor neurons and astrocytes in non-human primates after CSF infusion (Bevan et al., 2011; Samaranch et al., 2013; Passini et al., 2014). Presently it is unclear whether the differences in transduction profiles between mice and larger species is due to intrinsic differential tropism across species or technical challenges associated with intrathecal delivery in mice due to their small size. Nevertheless, Meyer at al. has recently shown that holding a non-human primate in the Trendelenburg position following an IT injection enhances AAV9 spread throughout the spinal cord and into the brain (Meyer et al., 2015). This type of injection is also feasible in the clinic, making this delivery technique relevant for clinical translation.

## Summary

Multiple studies have used AAVs to deliver therapeutic RNAi molecules (Table 1), but none reached the widespread gene transfer in CNS and periphery thought to be necessary to achieve transformative outcomes. Intravenous infusion of AAV9 in neonatal mice, such as in transduce peripheral muscle and mainly motor neurons in the spinal cord, but not in the cortex; while intravenous infusions in adults transduce mainly glial in the spinal cord. Lumbar intrathecal AAV injections in adult mice transduce local motor neurons in the spinal cord, but few if any in the cortex. Additionally, this type of delivery method has significant inter-animal variability. Intramuscular injections using vectors able to undergo retrograde transport transduce motor neurons, but only those neurons innervating the injected muscles. Lastly, neonatal intraventricular injections achieve widespread transduction of cortical and spinal cord motor neurons, as well as peripheral muscle, but gene expression is lost from dividing cells, such as astrocytes, which are known determinants of disease progression. Additionally, SOD1 reduction in upper or lower motor neurons alone is beneficial but not sufficient to substantially affect the course of disease progression. The many studies discussed here have clearly proven the potential of AAV-RNAi gene therapy for treating SOD1-ALS, and this approach is now also being applied to other ALS causative genes (Peters et al., 2015a), but more potent delivery vehicles are still needed to simultaneously target all disease-relevant CNS populations in adults. The recent improvements in injection techniques as well as development of engineered AAV capsids with vastly improved CNS transduction efficiency in adult animals are quickly paving the way to the emergence of potential transformative therapies for ALS and other neurodegenerative diseases (Choudhury et al., 2016; Deverman et al., 2016).

**Table 1:** **Preclinical viral vector mediated RNAi therapeutics for SOD1-ALS**.

**Animal model**	**Vector**	**Promoter**	**RNAi species (miR backbone)**	**Age at delivery**	**Total dose**	**Delivery method**	**Survival benefit**	**Study**
SOD1^*G*93*A*^ /B6/SJL mouse	VSV-G lentivirus	H1	shRNA	P40	180 ng of p24 antigen	IT, bilateral	None	Raoul et al., 2005
SOD1^*G*93*A*^ /B6/SJL mouse	Rabies-G EIAV lentivirus	H1	shRNA	P7	8.4E7−1.2E8 tu	IM, multiple muscle groups	77%	Ralph et al., 2005
SOD1^*G*93*A*^ /B6/SJL mouse	AAV6	H1	shRNA	P42	2E11 vg	IV	none	Towne et al., 2008
SOD1^*G*93*A*^ /B6/SJL mouse	AAV6	H1	shRNA	P1–P5, P15	3.7E8 tu	IM, multiple muscle groups	none	Towne et al., 2011
SOD1^*G*93*A*^ /B6/SJL mouse	AAV9	H1	shRNA	P1	5E11 vg	IV	39%	Foust et al., 2013
				P21	2E12 vg	IV	30%	
				P85	3E12 vg	IV	23%	
loxSOD1^*G*37*R*^ mouse				P215	3E12 vg	IV	22%	
SOD1^*G*93*A*^ /FVB/NJ mouse	AAVrh10	CBA	amiR (miR30a)	65	2.4e10 vg	IT	11%	Wang et al., 2014
SOD1^*G*93*A*^ /rat	AAV9	H1	shRNA	P70	1.6E11 vg	IC (motor cortex)	12%	Thomsen et al., 2014
SOD1^*G*93*A*^ /B6 mouse	AAV6	CMV	amiR (mir155)	P2	1.6E11 vg	ICV	26%	Dirren et al., 2015
	AAV9	GFAP	amiR (mir155)	P2	6.8E11 vg	ICV	14%	
	AAV6+AAV9	CMV/GFAP	amiR (mir155)	P2	8E10/3.4E10 vg	ICV	10%	
	AAV9	CMV	amiR (mir155)	P35	2.4E12 vg	IT	None	
	AAV9	GFAP	amiR (mir155)	P35	2.4E12 vg	IT	None	
SOD1^*G*93*A*^ /B6/SJL mouse	AAVrh10	CB	amiR (mir155) × 2	P56−68	2E11 vg	IV	20%	Borel et al., 2016
		U6	amiR (miR155)	P56−68	2E11 vg	IV	21%	
SOD1^*G*93*A*^ /B6/SJL mouse	AAV9	CBA	amiR (mir155) × 2	P1	1E11 vg	ICV	50%	Stoica et al., 2016

SOD1, superoxide dismutase 1; AAV, adeno associated viral vectors; VSV, vesicular stomatitis virus; IT, intrathecal; IM, intramuscular; IV, intravenous; IC, intracranial; ICV, intracerebral ventricular; P, postnatal day; tu, transducting units; vg, vector genomes.

## Author contributions

LS drafted the initial manuscript, and edited it into the final version. MSE edited the manuscript.

### Conflict of interest statement

The authors declare that the research was conducted in the absence of any commercial or financial relationships that could be construed as a potential conflict of interest.
